# Hidden burden of malaria in Indian women

**DOI:** 10.1186/1475-2875-8-281

**Published:** 2009-12-08

**Authors:** Vinod P Sharma

**Affiliations:** 1 Centre for Rural Development and Technology, Indian Institute of Technology, Hauz Khas, New Delhi-110016, India

## Abstract

Malaria is endemic in India with an estimated 70-100 million cases each year (1.6-1.8 million reported by NVBDCP); of this 50-55% are *Plasmodium vivax *and 45-50% *Plasmodium falciparum*. A recent study on malaria in pregnancy reported from undivided Madhya Pradesh state (includes Chhattisgarh state), that an estimated over 220,000 pregnant women contract malaria infection each year. Malaria in pregnancy caused- abortions 34.5%; stillbirths 9%; and maternal deaths 0.45%. Bulk of this tragic outcome can be averted by following the Roll Back Malaria/WHO recommendations of the use of malaria prevention i.e. indoor residual spraying (IRS)/insecticide-treated bed nets (ITN) preferably long-lasting treated bed nets (LLIN); intermittent preventive therapy (IPT); early diagnosis, prompt and complete treatment using microscopic/malaria rapid diagnostics test (RDT) and case management. High incidence in pregnancy has arisen because of malaria surveillance lacking coverage, lack of age and sex wise data, staff shortages, and intermittent preventive treatment (IPT) applicable in high transmission states/pockets is not included in the national drug policy- an essential component of fighting malaria in pregnancy in African settings. Inadequate surveillance and gross under-reporting has been highlighted time and again for over three decades. As a result the huge problem of malaria in pregnancy reported occasionally by researchers has remained hidden. Malaria in pregnancy may quicken severity in patients with drug resistant parasites, anaemia, endemic poverty, and malnutrition. There is, therefore, urgent need to streamline malaria control strategies to make a difference in tackling this grim scenario in human health.

## Background

A recent article on malaria in pregnancy (MiP) contributed by three prestigious organizations (Indian Council of Medical Research, National Vector Borne Disease Control Programme, World Health Organization), monitoring malaria situation in India has revealed appalling situation of pregnant women contracting malaria infection [1]. In undivided Madhya Pradesh (Madhya Pradesh and Chhattisgarh states of India), the estimated annual MiP was in excess of 220,000 infections; 76,000 abortions; 19,800 stillbirths; and 1,000 maternal deaths. In the past, several studies from India and other countries of the WHO's South East Asia Region have highlighted the tragedy of pregnant women living in malaria endemic regions [2]. Persistent neglect of MiP is widening the gender equity gap of the already skewed gender picture emerging due to social reasons [3]. It is noteworthy to mention that malaria incidence data reported by the NVBDCP is grossly under-reported, and this fact has been highlighted time and again by several independent studies and the NVBDCP's in-depth evaluations [4-7]. Furthermore, NVBDCP's surveillance does not collect data on malaria in pregnant women and children under five years of age (U5), and, principally for this reason, malaria in pregnancy has remained hidden [8]. Wherever and whenever quality surveillance was undertaken it has exposed the real picture of malaria. An account of the prevailing malaria situation of pregnant women, neonates, and U5 in India, and countermeasures to make a difference in the existing malaria situation in high-risk groups (pregnant women and U5), are briefly described in the discussion below.

## Discussion

Malaria is endemic in India, distributed throughout the length and breadth of the country, and an estimated >90% population (2001 population 1.13 billion) is at risk of the disease. Malaria surveillance is carried out at fortnightly intervals through a multi-purpose worker (MPWs) scheme at the village level, the active case detection (ACD). Blood film from fever cases are also collected at the Primary Health Centres (PHCs) and malaria clinics, called the passive case detection (PCD). In the last few years other health workers (e.g. Angarwari and Accredited Social Health Activist, ASHA) are also supplementing the blood smear collection from fever cases. Thus, NVBDCP collects about 100 million blood smears annually for <2 million parasite positive cases. ACD and PCD data is collated at the district and state level (state NVBDCP) and transmitted to NVBDCP for final compilation and decision-making. NVBDCP reported 1,785,109 parasite positive cases (9,44,741 Pv + 8,40,368 Pf) and 1,708 deaths in 2006. The 2007 data is still provisional [8]. The malaria situation in the country has remained static with small variations [8,9]. This unrealistic situation has arisen from the surveillance system that has broken down, and perhaps can only be relied on for malaria trends. Similarly malaria deaths are poorly recorded as the definition of death due to malaria require demonstration of malaria parasite in the peripheral blood, which is an unattainable goal in a highly diverse country with bulk of population in rural areas (c75%). As a result malaria has remained a huge problem in India with an estimated 90-160 million cases and more than 120,000 deaths in SEA Region [10], 70% of these from India. Furthermore, the existing surveillance system does not collect age and sex-wise data, abortions and still-births, which is vital for evaluating the impact of malaria in pregnancy [8]. Obviously, the problem of malaria in general and particularly in the high risk groups (pregnant women and U5) has remained hidden, although alarming as has been brought out by several independent studies [11-13]. The tragedy of being born in a poor and neglected settlements is revealed by the fact that hospital based studies in Ispat General Hospital in Rourkela, Orissa have revealed the rising trend of severity and multi-organ failure in *P. falciparum *infections [14]. Although the precise reasons for this paradigm shift in pathology is not understood, drug resistance may be an important contributing factor. The cost of treatment in the private sector and hospitalization is huge and unaffordable [14] in a country with 26% population living below poverty line (US cents c25/day) [15].

The distribution of malaria parasites viz., *P. vivax*, *P. falciparum *and *Plasmodium malariae *are highly uneven. Parasite formula changes from place to place and season to season, but can be interrupted by effective interventions. In general, the proportion of *P. vivax *is about 50% and *P. falciparum *around 40 to 45% for India; whereas *P. malariae *cases may be in thousands in forested areas of Orissa state (not recorded by the NVBDCP), and there is no focus of *Plasmodium ovale *and *Plasmodium knowlesi,* so far recorded [6,7]. Malaria in the north-eastern states is stable and in many other states with high proportion of *P. falciparum*, malaria is nearly stable; whereas in the rest of India it is unstable [16]. Malaria is entering towns and new territories under development slowly but steadily, e.g. towns in Orissa, Karnataka, Kerala etc. At present, rural malaria accounts for >90-95% cases and urban malaria <5-10% cases [17]. Low malaria incidence in urban areas is due to almost non-existing surveillance. The data is collected through the malaria clinics in hospitals. A retrospective study of data collected from leading hospitals in Ahmedabad revealed an average ratio of reported to estimated malaria cases as 36,766/4,119, i.e. nine times more than the reported rate, and 22 deaths per million population [6]. *Plasmodium falciparum *incidence could be very high in towns as for example, taken average of data between 1995-2000; Chennai represented 77% malaria cases and 61% of *P. falciparum *cases in Tamil Nadu state.

The strategic direction for controlling malaria in pregnancy has been well documented in several WHO publications. Pregnant women suffer from malaria on account of their change in body physiology making them at least twice as attractive to mosquitoes compared to non-pregnant women [18]. Any of the two parasites *P. vivax *or *P. falciparum*, or both are potentially dangerous infections in pregnancy and may produce fatal outcome to the mother or may lead to still birth, abortion, low birth weight (LBW) babies. In pregnancy, women have high proportion of *P. falciparum *infection, which is a potentially fatal disease [11-13,19,20]. There are compounding factors to the already bad malaria situation, particularly in pregnant women and their neonates. These are the mono- and multi-drug resistance in *P. falciparum *[21,22], and now reports of severe malaria in *P. vivax *cases [23], and chloroquine resistance in *P. vivax *[23]. Some anti-malarial drugs are contraindicated in pregnancy and in U5, due to G6PD deficiency e.g. primaquine, or artemisinin-based drugs [24,25]. An estimated 370 million populations (c25%) live below poverty line (BPL), and poverty worsen malaria situation. Studies in India have revealed that states with BPL population below national average of 26.1% have performed better in malaria control, than those with high BPL population [15]. Severe malaria, which is more frequent in MiP, requires hospitalization. Treatment cost of severe malaria is invariably far beyond affordable means of a common man, say USD 1,000 or more in private sector; and even in government hospitals (treatment is free), the cost of medicines has to be borne by the patients [14]. According to the National Family Health Survey III (2005-06), anaemia in India is alarmingly high. NFHS III reported that 79.1% of India's children between the ages of three and six, and 56.2% of married women in the age-group 15-49 were anaemia in 2006 [26]. Malaria in the background of endemic anaemia [27] could be a precipitating factor for complications during pregnancy. An epidemic of malaria in Mewat (Haryana) in 1977 in the background of endemic anaemia created chaos with protracted illness and deaths [28]. *Plasmodium falciparum *is also the most predominant infection in the tribal areas. A study in 1966 revealed that India's tribal population constitutes 7.8%, and this population contributes India's 40% malaria and 60% *P. falciparum *annually. This was the basis of a focused attack on the disease through World Bank Assistance in 1,045 PHCs in 108 districts of the eight states [29]. In addition to overall deteriorating malaria trends, malaria epidemics visit two or three endemic states annually. In epidemic situations, drug resistance strains abound [29]. All these add up to a dismal picture of malaria in pregnancy.

WHO recommends a three-pronged strategy that comprises: (i) intermittent preventive treatment (IPT) [30]; (ii) insecticide-treated nets, preferably long-lasting nets (LLINs) [31]; and (iii) case management of malaria illness [32]. Drug Policy of India, 2008 [26] is not aligned with the evidence-based best practices recommended by the Roll Back Malaria (RBM) initiative. IPT in pregnant women is recommended by WHO as an important component of RBM specifying epidemiological situations, although such situations exist in India and in the other countries of the South East Asia [30]. Most malaria deaths in pregnancy occur at home or in the private sector. Hard to reach, remote and difficult villages continue to suffer intensely from neglect requiring further decentralization of health care. Malaria prevention by indoor residual spraying DDT (Dichlorodiphenyl trichloroethane), Malathion and synthetic pyrethroids) has been almost given up in the country except under special situations [33]. NVBDCP is now focusing on insecticide-treated bed nets (ITN). In 2002-2003, 60,000 ITN and 1.2 million ITN in 2003-2004, were distributed in high malaria endemic districts, and, in 2005-2008, 4.2 million ITN were distributed in 10 states financed by the Global Fund [9]. For example, in-depth review of NVBDCP based on sampling of households revealed that ownership of bed nets was low, e.g. in high malarious states with proportion of households with at least one net and proportion with at least one treated net was 31.5% and 20.7% in Maharashtra, 29.3% and 17.5% in Orissa and 42.6% and 0.4% in Assam. Spray coverage of the target population varied from 35-57%, but the quality of households with uniform and complete coverage varied from 1.2 to 17.7% [33]. Table 1 gives the population under various categories as classified by the NVBDCP. High-risk populations requiring preventive malaria control represents 69 million, i.e. 5.78 million in NE and 63.22 million in EMCP states. These numbers are huge and its coverage requires a more realistic planning and mobilization of resources.

**Table 1:** Malaria in India in 2007

States	Population (2001 census in millions)	Malaria Positives (% +s out of total)	Pregnant Women In millions (2.9%)*	Children Under 5 in millions (12.1%)*	P. falciparum Positives (%)	Malaria deaths (% of total deaths)
NE States	38.49 (3.74%)	161,407 (10.9%)	1.12	4.66	120,871 (74.9% Pf)	494 (42.11%)

EMCP states	424.33 (41.32%)	990,575 (67.08%)	12.31	50.91	550,561 (55.58% Pf)	502 (42.79%)

Other states	462.82 (45.06%)	324,580 (21.98%)	13.42	56.00	65,417 (20.15% Pf)	177 (15.08%)

All India	1027 (100%)	1,476,562 (100%)	26.85	111.57	736,849 (49.9% Pf)	1173 (100%)

Census 2001 population data.

Enhanced Malaria Ccontrol Project (EMCP) states: Andhra Pradesh, Chhatisgarh, Gujarat, Jharkhand, Madhya Pradesh, Maharashtra, Orissa, Rajasthan

NE states: Arunachal Pradesh, Nagaland, Manipur, Mizoram, Tripura, Meghalaya, Assam.

*Based on WHO SEARO fact sheet [40].

Unfortunately bed nets distributed till the middle of 2006 have already lost their usefulness and require replacement as per the WHO norms (three-year life of nets) [30]. In early diagnosis, prompt and complete treatment NVBDCP is still depending on microscopic examination of blood smears (in 2006, 106,606,707 blood smears were examined and 1,785,109 were found malaria positive), but in Global Fund-financed districts malaria rapid diagnosis test (RDT) have been introduced. Management of *P. falciparum *has become a formidable challenge particularly in pregnancy [9]. High *P. falciparum*-states require priority attention to combat malaria in pregnancy through time-tested technologies recommended by WHO. Inertia would impinge on the national commitment of Millennium Development Goals (MDG) of halting the spread of malaria and reversing the trend, and Maternal Mortality Ratio (MMR), which is currently at 407 per 100,000 to 100 per 100,000 live births by 2015 [34]. Further, NVBDCP needs to keep up-to-date with new developments in malaria control. These are the intermittent preventive therapy for infants (IPTi) [35], rectal artesunate [36], strengthening small hospitals [37], new drugs in pipe line from Medicines for Malaria Venture (MMV)[38], and developments in Integrated Vector Management [39].

## Conclusion

Malaria in pregnancy has emerged as a major hidden public health problem at the national level. The following actions may be initiated for the management of malaria in pregnancy. These are the (i) intensive campaign to reduce anaemia on priority by up-scaling diagnosis and treatment of illnesses, birth control, supplementation of nutrition prioritizing areas with intense malaria transmission, (ii) recognition of malaria in pregnancy as an important health problem in India, its delimitation, stratification, and realistic calculations for planning and interventions; (iii) strengthening malaria surveillance and laboratory services for reliable data collection, age and sex-wise distribution, and other fatal outcome, in time and space; (iv) urgent need of scaling-up for impact of the preventive methods; (v) review of national malaria drug policy to include intermittent preventive therapy in pregnancy in stratified regions, similar to African settings; (vi) adoption of the RBM WHO recommendations on management of malaria and its control in pregnancy; (vii) care of illness in pregnancy; (viii) training of various categories of health staff; (ix) timely procurement of supplies, distribution, monitoring and evaluation; and (x) the programme should keep abreast with the new emerging technologies suitable for various epidemiological situations e.g. new drugs and improved drug delivery.

## Abbreviations

ACD: (Active case detection); ASHA: (Accredited social health activist); BPL: (Below poverty line); c: (About); EMCP: (Enhanced malaria control project); G6PD: (Glucose 6 phosphatase deficiency); IPT: (Intermittent preventive treatment); IPTi: (Intermittent preventive treatment infants); ITN: (Insecticide-treated bed nets); LBW: (Low birth weight); LLIN: (Long-lasting insecticide-treated nets); MiP: (Malaria in pregnancy); MMV: (Medicine for Malaria Venture); NE: (Northeastern states of India); NVBDCP: (National Vector Borne Disease Control Programme); PCD: (Passive case detection); RBM: (Roll Back Malaria); RDT: (Rapid diagnostics test); SEARO: (South East Asia Regional Office); U5: (Children under 5 years); WHO: (World Health Organization).

## Competing interests

The author declares that they have no competing interests.
